# Evaluating Functional Outcomes of Botulinum Toxin Type A Injection Combined with Occupational Therapy in the Upper Limbs of Children with Cerebral Palsy: A 9-Month Follow-Up from the Perspectives of Both Child and Caregiver

**DOI:** 10.1371/journal.pone.0142769

**Published:** 2015-11-24

**Authors:** Yu-Ching Lin, Chien-Yu Huang, I-Ling Lin, Jeng-Yi Shieh, Yu-Ting Chung, Kuan-Lin Chen

**Affiliations:** 1 Department of Physical Medicine and Rehabilitation, National Cheng Kung University Hospital, College of Medicine, National Cheng Kung University, Tainan, Taiwan; 2 Department of Physical Medicine and Rehabilitation, College of Medicine, National Cheng Kung University, Tainan, Taiwan; 3 Medical Device Innovation Center, National Cheng Kung University, Tainan, Taiwan; 4 School of Occupational Therapy, College of Medicine, National Taiwan University, Taipei, Taiwan; 5 Department of Medical Laboratory Sciences and Biotechnology, Kaohsiung Medical University, Kaohsiung, Taiwan; 6 Department of Physical Medicine and Rehabilitation, National Taiwan University Hospital, National Taiwan University, Taipei, Taiwan; 7 School of Occupational Therapy, College of Medicine, National Cheng Kung University, Tainan, Taiwan; Institute Pasteur, FRANCE

## Abstract

**Objective:**

To assess the effectiveness of combining botulinum toxin type A (BoNT-A) with functional occupational therapy (OT) at 9-month follow-up in children with cerebral palsy (CP) with bilateral upper limb impairments from the perspectives of both child and caregiver.

**Methods:**

Twelve children with CP and their caregivers were assessed across 5 time points over 9 months based on the ICF after BoNT-A injection and functional OT in this open-label study.

**Results:**

Significant differences were found across the 5 time points (*p* < .05) for both grasp and visual-motor integration with small effects (effect sizes = 0.12–0.24) and the self-care capability and performance of social function (*p <* .05). However, based on the effect sizes (0.02–0.14), no significant effects were found at the 4 post-test time points. Small effects were found on the psychological domain (effect sizes = 0.25–0.37) and environmental domains (effect size = 0.27) at follow-ups.

**Conclusion:**

Combining a BoNT-A injection with OT not only reduced the muscle tone and increased ROM but also improved the upper limb function and self-care capability in children with CP. More importantly, these effects persisted for up to 9 months. Functional OT extends the effectiveness of a BoNT-A injection.

## Introduction

Cerebral palsy (CP) is a group of permanent disorders of the development of movement and posture that are attributed to non-progressive disturbances occurring in the developing fetal or infant brain [1]. Its prevalence is 1.5 to 2.5 per 1,000 live births [2–4], and it is the most common cause of long-term disability in children [1,3]. Spastic type CP comprises about 80% of reported cases [5]. Spasticity has a primary impact on neurophysiological problems, such as abnormal muscle tone, primitive reflexes, movement, and/or postural control responses, and also causes secondary musculoskeletal problems, such as progressive changes in connective tissue, muscles and bones [6]. Furthermore, spasticity in the upper limbs particularly impairs independence in daily activities in children with spastic CP. Several treatments targeted at reducing muscle tone in the upper limbs to improve daily independence have evolved over time, one being botulinum toxin type A (BoNT-A) injection [7].

The efficacy and safety of the use of BoNT-A on the upper limbs have been established in children with CP [8,9]. Children’s responses to the treatment with BoNT-A are influenced by the formulation, dosage, appropriate administration, and subsequent rehabilitation programs after the intervention [10,11]. Evidence from meta-analysis and systematic review also supports the use of BoNT-A as an adjunct to occupational therapy (OT) to promote the function of the upper limbs of children with CP [12–14]. BoNT-A in conjunction with OT may extend the effectiveness of BoNT-A on decreasing spasticity at the body function level to execution of manual activity and participation in daily life at the levels of activity and participation based on the World Health Organization (WHO)’s international Classification of Functioning, Disability, and Health (ICF) [15,16]. Therefore, to comprehensively understand the efficacy of BoNT-A combined with OT, it is essential to evaluate the outcomes, from body function to activity and participation.

Previous studies of BoNT-A combined with OT in the upper limbs of children with CP have had three main limitations as follows. First, most of the evidence has been from children with unilateral CP. Hoare et al. suggested that further research is warranted for children with bilateral upper limb impairments [11]. Second, most studies have followed the effectiveness of BoNT-A injection in an upper limb for less than six months. However, according to our clinical observations, the effectiveness may persist for longer, up to 9 months, and for even more than 1 year, especially when the children receive OT functional programs after BoNT-A injection [17]. Third, the outcome measures used in previous studies focused only on children’s body functions and structures, without addressing children’s activity and participation or taking caregivers’ health-related quality of life (HRQOL) into account. However, from the viewpoint of holistic health care, outcome measures should be comprehensive and focus not only on clients’ impairments but also on their function. In addition, caregivers may have better HRQOL when their children have reduced spasticity and improved function in daily activity and participation.

Therefore, to fill the above gap, we recruited children with bilateral upper limb impairments and assessed the effectiveness of BoNT-A combined with functional OT with a 9-month follow-up in terms of children’s body functions and structures, activity, and participation, as well as caregivers’ subjective HRQOL according to the ICF. We hypothesized that children would have reduced spasticity, larger range of motion, and better upper limb function and daily participation, and that caregivers would have better HRQOL in the physical and psychological domains.

## Methods

### Participants

We recruited children between 3 and 12 years of age with confirmed diagnoses of cerebral palsy from a tertiary medical center. Inclusion criteria were: (1) diagnosis of spastic CP with bilateral upper limb impairments by pediatric neurologists or physiatrists, (2) moderate to severe spasticity over the upper limbs (Modified Ashworth Scale, MAS score≧2), (3) ability to extend the wrist greater than 20° and the range of motion (ROM) at finger metacarpophalangeal joints greater than 10° from full flexion, (4) ability to grasp a 3*3*3 centimeter cube, (5) no medication with anti-spasticity drugs, and (6) written informed consent from caregivers. Exclusion criteria included fixed contractures of the upper limbs, BoNT-A injections within 6 months, previous musculoskeletal surgery on the upper limbs, or contraindications to BoNT-A.

### Measures of child functioning

#### Range of motion (ROM) and spasticity

A goniometer and the MAS were used to assess passive ROM and spasticity of the affected upper limbs and lower limbs, respectively. The ROMs of the elbow, forearm, and wrist were measured. The MAS measures the resistance to passive movement of the muscles. Scores of the MAS range from 0–5[18]. A score of 0 indicates no resistance, and a score of 5 indicates rigidity.

#### Upper limb function

The Peabody Developmental Motor Scales, Second Edition (PDMS-2) was used to assess upper limb function. The PDMS-2 is a standardized, norm-referenced test consisting two composites, gross motor (reflexes, stationary, locomotion, and object manipulation) and fine motor (grasping and visual-motor integration). The fine motor composite of PDMS-2 is used to evaluate children’s upper limb function. Grasping is used to assess the ability to use the hands, and visual-motor integration is used to assess the ability to use visual perpetual skills to perform complex eye-hand coordination tasks. The psychometric properties of the PDMS-2 have been examined in children with CP [19]. The composite scores on the PDMS-2 have good test-retest reliability (intraclass correlation coefficients = 0.88–1.00). The sensitivity-to-change coefficients range from 1.6 to 2.1, and the responsiveness coefficients ranged from 1.7 to 2.3[19].

#### Activity and participation in self-care, mobility and social function

The Chinese version of the Pediatric Evaluation of Disability Inventory (PEDI-C) was used to assess children’s activity and participation in self-care, mobility, and social function in this study. The PEDI-C contains three scales, the Functional Skills Scale (FSS), the Caregiver Assistance Scale (CAS), and the Modifications Scale, and each includes three subdomains: self-care, mobility, and social function. The FSS and the CAS were used in this study to respectively assess children’s capability and performance on the subdomains of self-care, mobility, and social function. The PEDI-C, when used in children with CP, shows excellent internal consistency, test-retest reliability, concurrent validity, and discriminative validity [20].

### Measures of caregiver functioning

#### Health-related Quality of life (HRQOL)

The brief form of the World Health Organization Quality of Life scale (WHOQOL-BREF) was used to assess the HRQOL in caregivers of children with CP. The WHOQOL-BREF contains 26 universal items rated on a 5-point scale. A higher total score indicates better HRQOL. The reliability and validity of the Chinese version of the WHOQOL-BREF have been established [21]. The internal consistency ranges from 0.70 to 0.77 for the four domains. The test-retest reliability coefficients range from 0.41 to 0.79 at the item level and 0.76 to 0.80 at the domain level. Content validity coefficients are in the range of 0.53–0.78 for item-domain correlations and 0.51–0.64 for inter-domain correlations [21].

### Procedures

The study protocol was approved by the institutional review board of a medical center. Children and their caregivers were recruited from the outpatient department of our hospital. Parents signed informed consent forms before enrollment of their children. The first author administered the BoNT-A injections to all participants. EMLA cream 5% (2.5% lidocaine and 2.5% prilocaine, AstraZeneca Inc., Taiwan) was applied for topical anesthesia of the skin over the target muscles 60 minutes prior to the intramuscular administration of Botox (onabotulinumtoxinA, Allergan Inc., Irvine, CA, USA). One hundred units of Botox were reconstituted in 1 mL of normal saline. The total dosage of a Botox injection for each child was 10–15 units/kg/body weight, based on severity of spasticity, body weight, muscle size, and number of muscles injected, according to the recommendations in the published literature [8].

After the BoNT-A injection, children received OT two to three times a week for three months. Each therapy session of 30 minutes incorporated activities for strengthening the upper limbs and developing upper limb function in daily activities.

The children were evaluated with the ROM, the MAS, and the PDMS-2, and the caregivers were interviewed with the PEDI-C and completed the WHOQOL-BREF, at baseline and at 4 time points after the BoNT-A injections (1 month, 3 months, 6 months, and 9 months).

### Statistical analysis

Descriptive statistical analyses were conducted to characterize the basic properties of the observed variables. The hypotheses with respect to the PDMS-2, the PEDI-C, and the WHOQOL-BREF were analyzed using 1-tailed repeated-measures analysis of variance (group [1] X time [5]) (data in S1 Dataset). The probability of Type I errors (alpha) was determined as .5. If the sphericity assumption was not met for the variables, degrees of freedom for the F ratio were adjusted according to the Greenhouse Geisser epsilon. Effect sizes at the four follow-up time points were also calculated using the difference scores divided by the standard deviation of the pretest score.

## Results

Descriptive statistics of the participants are shown in Table 1. A total of 12 children with CP (4 boys and 8 girls) and their caregivers were recruited in the study. The mean age of the children was 7.60 years (SD: 2.42). All children were spastic type. They had spasticity over bilateral lower limbs and at least one upper limb. Half of the children were ambulatory (Gross Motor Function Classification System, GMFCS level II and III), and a majority of the patients (n = 9) had difficulties in manipulating objects (Manual Ability Classification System, MACS levels III to V).

**Table 1:** Characteristics of the participants (N = 12).

Variables	Statistics
Children	
Age: mean (SD)	7.6 (2.4)
Sex (male/female): n (%)	4 (0.3) / 8 (0.7)
GMFCS^a^/MACS^b^: n (%)	
I	1 (8.3) / 0 (0.0)
II	4 (33.3) / 3 (25.0)
III	1 (8.3) / 3 (25.0)
IV	4 (33.3) / 4 (33.3)
V	2 (16.7) / 2 (16.7)
Caregiver	
Age: mean (SD)	36.3 (4.4)
Sex (female/male): n (%)	10 (80.0) / 2 (20.0)
Respondent (mother/father/other): n (%)	9 (70.0) / 2 (20.0) / 1 (10.0)

^a^ GMFCS: Gross Motor Function Classification System

^b^ MACS: Manual Ability Classification System

The distribution and severity of spasticity in the upper limbs and lower limbs for each child are detailed in Table 2. In addition, Table 3 contains the information of Botox dose injection for individual muscles and the average Botox dose per muscle group per kilogram of body weight. Child number 11 only received BoNT-A injection for spasticity of his upper limbs due to budget limitations. Table 4 shows the changes in ROM and spasticity from the baseline at the 1-month, 3-month, 6-month, and 9-month follow-ups. Children’s ROM improved and the effect persisted to 9 months after the BoNT-A injection. Patients’ muscle tone decreased and the effect persisted to 9 months after the BoNT-A injection.

**Table 2:** The spasticity distribution of muscles for the botulinum toxin type A injection for each child.

Muscle	Side^a^	Participants: Modified Ashworth scale											
		1	2	3	4	5	6	7	8	9	10	11	12
Arm													
Biceps brachii	L				3	4							
Forearm													
Pronator teres	L				3	4	2	3					
Pronator teres	R		3									3	
Pronator Quadratus	R	2										3	
Wrist													
Flexor carpi ulnaris	L			3	4	4	3	4		3		4	
Flexor carpi ulnaris	R	3	3						3		2	4	2
Flexor carpi radialis	L						3						
Thumb													
Thumb adductors							2						
Flexor pollicis Brevis							2						
Thigh													
Hip adductors	L				4								
Hip adductors	R				4								
Leg													
Knee flexors	L			4		4							
Knee flexors	R			3		4							
Gastrocnemius	L	4	4	4	4	4	3	4	4	4	3		3
Gastrocnemius	R	4	4	3	4	4			4	4	3		4
Soleus	L						3			3			
Soleus	R									3			

^a^ L = Left; R = Right

**Table 3:** The dosage of botulinum toxin type A (Botox, onabotulinumtoxinA) injection in each muscle for each child.

Muscle	Side^a^	Participants: dose												Average dose per kilogram body weight
		1	2	3	4	5	6	7	8	9	10	11	12	
		22 kg	20 kg	20 kg	13 kg	12 kg	16 kg	15 kg	16 kg	22 kg	14 kg	30 kg	20 kg	
Arm														
Biceps brachii	L				10	10								0.80
Forearm														
Pronator teres	L			30	20	10	40	65		40		60		2.07
Pronator teres	R	50	60						30		30	80	30	2.24
Pronator Quadratus	L						30							1.88
Wrist														
Flexor carpi ulnaris	L				10	10	30	30						1.37
Flexor carpi ulnaris	R		50									30		1.75
Flexor carpi radialis	R	50										30		1.64
Thumb														
Thumb adductors	L						15							0.94
Flexor pollicis Brevis	L						15							0.94
Thigh														
Hip adductors	L				50									3.85
Hip adductors	R				50									3.85
Leg														
Knee flexors	L			70		30								3.00
Knee flexors	R			60		30								
Gastrocnemius	L	100	60	75	30	40	80	105	30	100	50		70	3.86
Gastrocnemius	R	100	60	65	30	40			30	100	50		130	3.66
Soleus	L						30			30				1.62
Soleus	R									30				1.36
Total dose injected		300	230	300	200	200	240	200	90	300	130	200	230	

^a^ L = Left; R = Right

**Table 4:** ROM and spasticity changes from baseline after botulinum toxin type A injection.

	Baseline mean (SD)	1 month mean (SD)	3 months mean (SD)	6 months mean (SD)	9 months mean (SD)
ROM^a^					
Elbow (n = 2)	65.0 (7.1)	+55.0 (21.2)	+55.0 (21.2)	+55.0 (21.2)	+50.0 (28.3)
Forearm (n = 12)	94.2 (32.3)	+28.3 (19.5)	+26.7 (19.2)	+26.7 (19.2)	+21.7 (17.0)
Wrist (n = 7)	78.6 (23.4)	+24.3 (21.5)	+21.4 (18.6)	+14.3 (29.9)	+18.6 (19.5)
Modified Ashworth scale					
Biceps brachii (n = 2)	3.5 (0.7)	-2.5 (0.7)	-2.0 (0.0)	-2.0 (0.0)	-1.0 (0.0)
Pronators (n = 12)	3.2 (0.7)	-1.4 (0.7)	-1.3 (0.8)	-1.4 (0.5)	-1.1 (0.5)
Wrist flexors (n = 7)	2.9 (0.7)	-1.7 (0.8)	-1.6 (0.8)	-1.1 (0.9)	-1.3 (0.8)
Adductor of thumb (n = 1)	2.0 (0.0)	-1.0 (0.0)	-1.0 (0.0)	-1.0 (0.0)	-1.0 (0.0)
Flexor pollicis brevis (n = 1)	1.0 (0.0)	-1.0 (0.0)	-1.0 (0.0)	-1.0 (0.0)	-1.0 (0.0)

^a^ ROM: Range of Motion

Table 5 shows the mean scores of the PDMS-2, the PEDI-C, and the WHOQOL-BREF and their corresponding SDs at the 5 time points, and the effect sizes at the 4 post-test time points. For both grasp and visual-motor integration subscales of the PDMS-2, significant differences were found across 5 time points (*p* < .05). Children improved on grasp and visual-motor integration after the BoNT-A injection, and these effects persisted for up to 9 months. In addition, small effects were found at 3, 6, and 9 months on both grasp and visual-motor integration subscales (effect sizes = 0.12–0.24).

**Table 5:** The mean scores of the PDMS-2, the PEDI-C, and the WHOQOL-BREF at 5 time points and the effect sizes at 4 post-test time points (N = 12).

Child Functioning	Pretest	1 month		3 months		6 months		9 months		*p* value
	Mean (SD)	Mean (SD)	ES^a^	Mean (SD)	ES	Mean (SD)	ES	Mean (SD)	ES	
PDMS-2^b^										
Grasp	34.08 (15.37)	35.17 (15.87)	0.08	37.75 (15.40)	0.24	36.83 (15.50)	0.18	37.75 (16.06)	0.24	0.03
Visual-Motor Integration	88.58 (43.69)	94.00 (45.88)	0.12	97.92 (46.14)	0.21	98.75 (45.87)	0.23	99.50 (44.34)	0.12	0.03
PEDI-C^c^										
FSS^d^-self care	40.67 (17.53)	42.08 (18.23)	0.08	42.75 (18.58)	0.11	43.17 (18.65)	0.14	43.08 (18.92)	0.14	0.03
FSS-mobility	28.17 (19.80)	28.58 (20.09)	0.02	29.33 (19.76)	0.06	29.67 (20.18)	0.07	29.67 (20.30)	0.07	0.09
FSS-social function	47.00 (13.82)	47.50 (14.07)	0.04	47.92 (14.25)	0.07	48.08 (13.85)	0.08	48.00 (13.89)	0.07	< .001
CAS^e^-self care	16.50 (08.39)	17.17 (08.78)	0.08	17.42 (08.84)	0.11	17.25 (09.02)	0.09	17.33 (08.94)	0.10	0.11
CAS-mobility	14.92 (12.61)	15.67 (12.89)	0.06	15.75 (12.82)	0.07	15.83 (13.03)	0.07	15.92 (13.20)	0.08	0.32
CAS-social function	12.73 (06.07)	12.91 (06.14)	0.03	13.18 (06.31)	0.07	13.55 (06.27)	0.14	13.36 (06.22)	0.11	0.02
Caregiver functioning	Pretest	1 month		3 months		6 months		9 months		
	Mean (SD)	Mean (SD)	ES	Mean (SD)	ES	Mean (SD)	ES	Mean (SD)	ES	*P* value
WHOQOL-BREF^f^										
Physical	14.48 (0.74)	14.76 (0.84)	0.11	14.29 (0.64)	-0.07	14.33 (0.69)	-0.05	14.33 (0.73)	-0.05	0.09
Psychological	12.33 (0.83)	13.33 (0.78)	0.35	13.39 (0.72)	0.37	12.67 (0.68)	0.11	13.06 (0.69)	0.25	0.16
Social	14.33 (0.62)	14.11 (0.60)	-0.10	14.00 (0.51)	0.15	13.78 (0.50)	-0.26	14.00 (0.63)	-0.15	0.55
Environmental	12.75 (0.53)	12.92 (0.67)	0.09	12.83 (0.60)	0.04	13.25 (0.50)	0.27	12.67 (0.66)	-0.04	0.52

^a^ ES: Effect Size

^b^ PDSM-2: Peabody Developmental Motor Scales, Second Edition

^c^ PEDI-C: Chinese version of the Pediatric Evaluation of Disability Inventory

^d^ FSS: Functional Skill Scale

^e^ CAS: Caregiver Assistance Scale

^f^ WHOQOL-BREF: World Health Organization Quality of Life

For the PEDI-C, significant differences were found on the self-care domain of the FSS (*p* = 0.03), and on the social domain of both the FSS and the CAS (*p < .05)*. However, based on the effect sizes (0.02–0.14), no significant effects were found at the 4 post-test time points. For the WHOQOL-BREF, no significant differences were found for the four domains (*p* > .05), but small effects were found on the psychological domain at the 1-month, 3-month, and 9-month follow-ups (effect sizes = 0.25–0.37). Small positive effects were found on the environmental domain at the 6-month follow-up (effect size = 0.27), while the social domains showed a negative effect at the 6-month follow-up (effect size = -0.26).

All participants tolerated the injection procedures well. There were no side effects noted during the administration of Botox or at the various follow-up points after the treatment.

## Discussion

The present study is the first prospective study to explore the effectiveness of a BoNT-A injection and functional OT in children with bilateral upper limb impairments up to 9 months from the perspectives of both child and caregiver. We found that a BoNT-A injection combined with OT not only reduced the muscle tone and increased ROM but also improved the upper limb function and self-care capability in children with CP. More importantly, these effects could persist for as long as 9 months. Functional OT programs may extend the effectiveness of a BoNT-A injection.

Our findings are in line with the hypotheses that a combination of BoNT-A and OT is more effective than OT alone for children with spastic CP in the randomized control trial by Wallen et al. [22] and the comprehensive systemic review by Hoare et al. [11], and ours explored the possibility that the effectiveness can be extended to 9 months. This finding encourages the use of routine OT functional programs after a BoNT-A injection to facilitate children’s upper limb function when their spasticity is better controlled by the BoNT-A injection. In addition, since the effects of the BoNT-A injection could last for 9 months when combined with functional OT programs, the duration between two adjacent injections could possibly be postponed to 9 months or longer. Such a postponement would decrease the frequency of injections and lessen the financial burden on the families of children with CP.

In an animal model study for time course of recovery of rat juvenile skeletal muscle after BoNT-A injection, Ma et al. found that muscle mass, electrophysiologic variables, and muscle force generation returned to nearly normal at 6 months postinjection [23]. They suggested intervals of three to six months between two sessions of BoNT-A injections in clinical situations. The mean duration of the effect for BoNT-A in the lower limbs of children with CP from the majority of clinical trials was three to four months [24]. Therefore, the effects for BoNT-A in the upper limbs were usually followed for less than 6 months in previous studies [11]. The duration of the effects of the BoNT-A injection on the upper limbs in children with CP is reported to range from 8 weeks to 6 months in the literature [11]. The various durations may have been influenced by differences in adjunct therapy, severity of spasticity, characteristics of recruited participants, and the dosage of BoNT-A [8,11,14].

Lannin et al. conducted a survey of OT and physical therapy practice for children with CP after BoNT-A intervention [25]. They found that most occupational therapists and physical therapists did not particularly focus on achieving functional outcomes related to the intervened muscles and their function. Our practice protocol required occupational therapists to develop OT programs for the affected upper limbs in a clinical setting and in a daily context for our participants following BoNT-A injection. According to the current evidence, task-oriented training may provide a better chance of motor learning and augment the effects of BoNT-A, including the prolonged duration of effect.

Our study is the first prospective trial to follow CP children with bilateral upper limb impairments until 9 months after injection with BoNT-A. This period was chosen because we found the effects of BoNT-A injection persisted longer in the upper limbs than in the lower limbs from our clinical experience. Fattal-Valevski et al. also had similar findings in their retrospective investigation [17]. They assessed the effects of two different types of botulinum toxin A injections, Dysport (AbobotulinumtoxinA) and Botox (OnabotulinumtoxinA), on upper limb spasticity in 30 consecutive children with CP. The mean doses were 294 ± 167 units for Dysport and 127 ± 50 units for Botox. Although these total dosages of BoNT-A were lower than the recommended dosages, the mean duration of the effects was 7 months (range, 1–12 months) and was longer than what had been reported in previous studies on lower limbs in children with CP. However, in the randomized controlled trial by Wallen et al., they administered the doses of Botox with 2 to 13 units/kg (mean, 8.1 ± 2.9 units/kg) to 40 children with CP to investigate the functional outcomes of the upper limb in four groups: BoNT-A plus OT, BoNT-A alone, OT alone, and a no-treatment control group [22]. The BoNT-A plus OT group made improvement at 3 months in the primary outcomes of Goal Attainment Scale (GAS) and Canadian Occupational Performance Measure (COPM) compared with other three groups. No differences in their functional outcomes were found among the four groups at 6 months. Significant reductions in muscle tone were noted at 2 weeks and 3 months after injection, the muscle tone returned to the baseline level at 6 months. The difference in dosage of BoNT-A, OT training strategies following intervention, and characteristics of participants may account for the different durations of effects in our and above two studies.

Table 3 denotes the dose of individual muscle for each participant and the mean dose for each injected muscle. Although the total dosage of 10–15 units/kg of Botox in our trial was higher than total dosage in study of Wallen et al. [22], the total dosage was distributed for injection of upper limbs and lower limbs rather than for only upper limbs. Indeed, the Botox doses for muscles of upper limb in our study ranging from 0.8 to 2.24 units/kg were relatively low as compared to the recommended dosage for upper limb in children with CP. In the trial of Wallen et al., the mean doses of Botox (units/kg) injected into muscles of the upper limbs were 4.3±0.8 for elbow flexors, 2.0±0.4 for pronators, 1.7±0.5 for wrist flexors, and 2.2±0.7 for the thumb [22]. Their doses for upper limbs were all higher than the doses administered to muscles of the upper limbs for our participants (Table 3). In their study, the BoNT-A plus OT group improved significantly at 3 months but did not differ at 6 months in the primary outcomes of GAS and COPM compared with other groups of BoNT-A alone, OT alone, and control group.

As regards the dosage of BoNT-A and the duration of effects, higher doses of BoNT-A injection theoretically may have a longer duration of effect. But there is no literature supporting that larger total doses distributed to the upper and lower limbs, as seen in our study, can extend the effects of the upper limbs. To our best knowledge, there is no reliable evidence on the relationship between dosage of BoNT-A and effect on function of upper limbs in human studies. The recommendations for total doses and individual doses for each muscle come from opinions or consensus of experts and a few clinical trials with small sample sizes [26]. Randomized, double-blind, placebo-controlled, dose-ranging studies for BoNT-A injection in children with cerebral palsy are especially rare [26]. In addition, tone reduction in muscles through the administration of BoNT-A does not necessarily result in corresponding improvement of function. As the dosage for upper limbs in our participants was not higher than the dosage used in the study of Wallen et al. [22], we believe that the elongated period of reduction of muscle tone and improved function of the upper limbs in our trial is more likely to have arisen from a synergistic effect of combining functional OT training and BoNT-A intervention on neuroplasticity [13].

The characteristics of the participants may also account for the different durations of effect after administration of BoNT-A. A retrospective chart review by Fattal-Valevski and his colleagues reported a mean effective duration after BoNT-A injection to an upper limb of 7.0 ± 3.0 months in 30 children with CP (mean age, 9.9 ± 5.0 years). Their patients were heterogeneous, including 21 with hemiplegia, 5 with triplegia, and 4 with quadriplegia [17]. In the trial of Wallen et al. [22], their 40 participants with CP were also heterogeneous, including spastic hemiparesis, spastic triplegia, and spastic quadriplegia. They stated that their sample size was too small for subgroup analysis to determine any differences in the responses of children with different types of CP. They also suggested that future studies specifically for children with triplegia and quadriplegia will be required to answer their responsiveness. Unlike those in the study by Fattal-Valevski et al. [17] and Wallen et al. [22], all patients in our study had CP with bilateral upper limb impairments, which may increase the generalizability of our results.

However, since insignificant differences in the self-care domain of the CAS were found across the 5 time points, it is noted that the daily capacity and participation of the children did not improve correspondingly, based on the improved upper limb function. Four possibilities might explain this lack of improvement. First, the progress in upper limb function and the self-care capability may not have been sufficient for the children to execute those daily activities with significant reductions of caregiver assistance. Therefore, their caregivers may still have managed the children’s daily activities. Second, the children may have been slow to accomplish the daily activities, despite improvements in their capability to do them. Due to the tight schedules of the daily lives of both the children and the caregivers, the caregivers may have tended to help the children deal with these daily activities. Third, the caregivers, being accustomed to assisting the children with executing daily activities, may have continued to provide the same amount of assistance. Even though the capabilities of the children may have improved, the caregivers may not have noticed and thus may not have provided children more opportunities to participate in their daily activities. Caregiver education in the clinic is recommended to help caregivers to provide assistance that corresponds to children’s capabilities in daily participation.

Last, the measure we used, i.e., the CAS of the PEDI-C, might not be responsive enough to reflect the changes of children’s performance in the self-care domain in our study. The CAS of the PEDI-C is rated on a 6-point scale of 0-total dependent, 1-maximal assistance (75–99%), 2-moderate assistance (25–75%), 3-minimal assistance (less than 25%), 4-supervision, and 5-independent. It is noted that point 2, i.e., moderate assistance, covers a wide range of assistance (25–75%). Subtle changes in children’s performance, such as the percentage of assistance decreasing from 75 to 60, might not detectable with the CAS of the PEDI-C. Moreover, most of the children (75%) in our study, whose MACS levels were from III to V, had high possibilities to have only subtle changes in self-care performance. As a result, due to the design of the CAS of the PEDI-C, it might be reasonable that there was no significant difference in the self-care domain of the CAS during the 9-month period.

Moreover, we found no significant differences in the HRQOL domains across the 5 time points. This result suggests that the improvement of children’s self-care capacity and upper limb function after a BoNT-A injection and functional OT programs might not be accompanied by changes in the caregiver’s HRQOL. Koman et al. examined the short-term outcomes of using BoNT-A for upper limb spasticity in children with CP with a randomized, double-blind, placebo-controlled design [7]. Similar to this present study, their study identified no significant differences in parents/caregivers’ HRQOL after BoNT-A or placebo injections. Moreover, some other studies also showed that caregiver’s HRQOL was not necessarily associated with children’s functioning [22], nor did it necessarily correspond to improvements in children’s functioning [23]. However, it is noted that the sample size in our exploratory prospective study was small, which restricts the interpretation of our study. More studies are warranted to validate the longitudinal impact of the BoNT-A injection combined with functional OT programs on caregivers’ HRQOL using a larger sample size.

Our results are consistent with the major conclusion from a recent systemic review that a combination of BoNT-A and OT is more effective than OT alone for children with spastic cerebral palsy in reducing impairment and improving activity level outcomes, but not for improving HRQOL [11]. We also provide evidence that the effects persisted for at least 9 months when BoNT-A injection was combined with functional OT in CP children with bilateral upper limb impairments.

However, this study still has three limitations that may concern readers: it was not a double-blind placebo-controlled (DBPC) study, the sample size was small, and the length of the study may have been insufficient. Due to the limited range of participant characteristics, the design combining two interventions, and the long follow-up of this study, we could not enroll more children or follow them for longer than 1 year due to limited resources. Future DBPC studies that recruit more participants and have longer follow-ups (e.g., 1 year) based on the randomized controlled design are warranted to examine the long-term effectiveness of combining BoNT-A injection with OT.

## Conclusion

In conclusion, our findings indicate that functional OT programs can extend the effectiveness of a BoNT-A injection to as long as 9 months. The combination of a BoNT-A injection with OT not only reduces spasticity and improves ROM in the upper limbs but also improves the function of the upper limbs and self-care capability of children with CP. However, children’s daily participation and caregivers’ HRQOL did not demonstrate corresponding improvements during the study period. Future DBPC studies that recruit more participants and have longer follow-up periods are warranted to examine the long-term effectiveness of the combination of BoNT-A injections and regular OT.

## Supporting Information

S1 Dataset Dataset used in this study. (SAV)
